# Complex and non-redundant signals from individual odor receptors that underlie chemotaxis behavior in *Drosophila melanogaster* larvae

**DOI:** 10.1242/bio.20148573

**Published:** 2014-09-19

**Authors:** Jeewanjot S. Grewal, Christine Nguyen, Raquel Robles, Christina Cho, Karolina Kir, Nicole Fledderman, George Gacharna, Michael Wesolowski, Christie Klinger, Pedro Vallejo, Lorien Menhennett, Abhiram Nagaraj, Chineze Ebo, Garrett Peacy, Eftihia Davelis, David Kucher, Sarah Giers, Scott A. Kreher

**Affiliations:** Department of Biological Sciences, Dominican University, 7900 West Division Street, Parmer Hall 244, River Forest, IL 60305, USA

**Keywords:** Odor receptors, Olfaction, *Drosophila*, Or42a, Or42b

## Abstract

The rules by which odor receptors encode odors and allow behavior are still largely unexplored. Although large data sets of electrophysiological responses of receptors to odors have been generated, few hypotheses have been tested with behavioral assays. We use a data set on odor responses of *Drosophila* larval odor receptors coupled with chemotaxis behavioral assays to examine rules of odor coding. Using mutants of odor receptors, we have found that odor receptors with similar electrophysiological responses to odors across concentrations play non-redundant roles in odor coding at specific odor concentrations. We have also found that high affinity receptors for odors determine behavioral response thresholds, but the rules for determining peak behavioral responses are more complex. While receptor mutants typically show loss of attraction to odors, some receptor mutants result in increased attraction at specific odor concentrations. The odor receptor mutants were rescued using transgenic expression of odor receptors, validating assignment of phenotypes to the alleles. Vapor pressures alone cannot fully explain behavior in our assay. Finally, some odors that did not elicit strong electrophysiological responses are associated with behavioral phenotypes upon examination of odor receptor mutants. This result is consistent with the role of sensory neurons in lateral inhibition via local interneurons in the antennal lobe. Taken together, our results suggest a complexity of odor coding rules even in a simple olfactory sensory system.

## INTRODUCTION

Odor coding is hypothesized to be combinatorial: a repertoire of odor receptors (*Or*) has differential responses to odors across concentrations, allowing detection and discrimination of odors. The combinatorial code hypothesis has been supported by analysis of odor receptors from multiple animals: in electrophysiological and imaging studies, both *in vitro* and *in vivo*, different odors elicit activity in distinct subsets of odor receptors (Malnic et al., 1999; Hallem and Carlson, 2006; Nara et al., 2011; Silbering et al., 2011). Furthermore, testing single odors across concentrations recruits different subsets of receptors, typically with more receptors becoming activated with increase of odor concentration (de Bruyne et al., 2001; Wang et al., 2003; Hallem and Carlson, 2006).

A remaining problem to address is to understand the relationship between odor receptor activity and behavior, particularly the rules by which an odor receptor repertoire allows behavioral responses to odors and discrimination of odors. For example, if an odor receptor uniquely responds to an odor, it is reasonable to hypothesize that the receptor is necessary for behavioral response to the odor, and evidence supports this hypothesis (Stensmyr et al., 2012; Mathew et al., 2013). However, if multiple receptors respond to an odor, which is the more common case, is each receptor functionally redundant, so that loss of a single receptor does not affect behavioral response to the odor?

A related problem is how to identify behaviorally relevant ligands for odor receptors: if an odor elicits strong activity from a receptor, is that response critical for a behavioral response? Likewise, if an odor elicits only a small or modest receptor response, is that signal irrelevant for the behavioral response to the odor? Furthermore, are the highest affinity receptors for an odor responsible for setting behavioral thresholds, or are sensory neurons weighted differentially?

Testing hypotheses on the rules of odor coding has been difficult due to the fact that most animals possess hundreds or thousands of odor receptor genes (Ache and Young, 2005). Insects have been especially attractive in studies on olfaction due to the observation that some insect genomes contain less than 100 odor receptor genes (van der Goes van Naters and Carlson, 2006). A well-studied insect odor receptor linked to behavior is the *Drosophila* carbon dioxide receptor. In *Drosophila* two receptor genes, *Gr63a* and *Gr21a*, are co-expressed in a single class of sensory neuron and are necessary and sufficient for reception of carbon dioxide (Jones et al., 2007; Kwon et al., 2007). *Gr63a* and *Gr21a* and their orthologs are also responsible for mediating behavioral responses to carbon dioxide in mosquitoes, and inhibition of this receptor may prevent host seeking in mosquitoes (Turner and Ray, 2009; Turner et al., 2011). The *Gr63a*/*Gr21a* receptor complex electrophysiologically responds in a dose-dependent manner to CO_2_ concentrations, and this encoded information is conveyed in two divergent pathways to the *Drosophila* brain to ensure proper behavioral responses across concentrations (Lin et al., 2013). However, unlike detection of other odors, carbon dioxide is detected by a single receptor complex within a single class of sensory neuron, and thus carbon dioxide coding is non-combinatorial.

What is needed is a system to test the role of two or more receptors in combinatorial coding of odors. The *Drosophila* larva is ideal for use in analysis of how odors are coded: the larva only uses ∼25 odor receptors in its olfactory sensory system and has 21 pairs of olfactory sensory neurons (Fishilevich et al., 2005; Kreher et al., 2005). In most cases, only one canonical odor receptor is expressed per olfactory sensory neuron pair (Fishilevich et al., 2005; Kreher et al., 2005). Further, the larval odor receptor repertoire has been comprehensively tested in terms of electrophysiological responsiveness to odors in the “empty neuron” system (Dobritsa et al., 2003; Kreher et al., 2008). Calcium imaging studies and direct recordings from larval olfactory sensory neurons have led to similar conclusions about the receptive ranges of odor receptors, supporting assignment of ligands to the larval odor receptor repertoire (Asahina et al., 2009; Hoare et al., 2011).

The overall anatomy of the larval olfactory neuronal circuit is similar to adult *Drosophila* and superficially similar to vertebrates. Larval olfactory sensory neurons make synapses in the larval antennal lobe within the larval brain (Fishilevich et al., 2005; Kreher et al., 2005; Stocker, 2008). Second order neurons, projection neurons, are post-synaptic to sensory neurons and transmit information to the higher brain regions, the mushroom bodies and lateral horns (Stocker, 2008). Olfactory sensory neurons also make synapses with local interneurons within the larval antennal lobe, which may allow filtering and gain control of incoming olfactory sensory information (Stocker, 2008; Asahina et al., 2009). Thus, the larval *Drosophila* olfactory system is an ideal blend of numerical simplicity and network complexity to test hypotheses of the combinatorial coding of odors.

Multiple behavioral assays have been used to characterize behavior in the *Drosophila* larva in response to multiple classes of odors (Cobb et al., 1992; Cobb and Dannet, 1994; Khurana and Siddiqi, 2013). Chemotaxis behavior has been characterized quantitatively in response to defined odor gradients of known steepness (Gomez-Marin et al., 2011; Gershow et al., 2012; Gomez-Marin and Louis, 2014). Tests of odor segmentation responses and discrimination through cross-generalization have also been used to define perceptions of odors (Boyle and Cobb, 2005; Gerber and Stocker, 2007; Kreher et al., 2008).

In this study, we have used the larval odor receptor data set to test hypotheses by examining larval chemotaxis behavior with the two-choice assay, which affords statistical power and allows analysis of a breadth of odor concentration gradients (Kreher et al., 2008; Mathew et al., 2013; Khurana and Siddiqi, 2013). The larval odor receptor data set contains responses of all functional larval odor receptors to a systematically tested odor panel at two concentrations (Kreher et al., 2008). While some preliminary behavioral hypotheses have been tested using the larval odor receptor response data set, key questions on the relationship between odor receptor activity and behavior have yet to be answered. We have found that receptors with similar electrophysiological responses to odors across concentrations show non-redundancy, but only at specific concentrations. We have also found that high affinity odor receptors set behavioral response thresholds, but that the rules governing peak responses are more complex. The odor receptor mutants were rescued using GAL4-UAS transgenic expression of odor receptors, validating assignment of phenotypes to the alleles. Variation in behavioral responses measured by the two-choice assay cannot be completely explained by vapor pressures of odor molecules. Finally, we have found that odor receptor mutant larvae have behavioral phenotypes in response to odors that are weak electrophysiological ligands, consistent with the role of olfactory sensory neurons allowing gain control of the olfactory circuit.

## MATERIALS AND METHODS

### Genetics

Flies were housed on standard cornmeal–dextrose–yeast medium (obtained from the University of Illinois–Chicago) at 25°C, on a 12 hour light–dark cycle. Mutants were obtained from the Bloomington Stock Center: *Or42a^f04305^*, *Or42b^EY14886^*, and *Or83b^2^*; *Or83b* has been renamed *Orco* and is referred to as such in this study. Both *Or42a* and *Or42b* are expressed in the larval and adult olfactory systems, and the loss-of-function mutants have been partially characterized previously (Kreher et al., 2008; Olsen et al., 2010; Nagel and Wilson, 2011; Mathew et al., 2013; Shim et al., 2013). Odor receptor mutant lines were backcrossed to a Canton-S line with a *w^−^* allele (wCS); the wCS line was used as the wild type control in behavioral assays.

Mutant rescue experiments were conducted by combining Odor receptor GAL4-UAS-Odor receptor lines. The *Or42a^−^* mutant was rescued with *Or42a*GAL4-UAS-*Or42a*; the *Or42b^−^* mutant was rescued with *Or42a*GAL4-UAS-*Or42b*. To generate large numbers of rescue larvae, *Or*GAL4 and UAS-*Or* lines were recombined onto the same third chromosome and then combined with the respective mutant alleles; genotypes are: *Or42a* rescue (*Or42a^−^*; *Or42a*GAL4-UAS-*Or42a*); *Or42b* rescue (*Or42b^−^*; *Or42b*GAL4-UAS-*Or42b*). The mutant controls were the mutant alleles combined with *Or42a*GAL4 and *Or42b*GAL4, which should display the mutant phenotype without rescue; genotypes are: *Or42a* mutant control (*Or42a^−^*; *Or42a*GAL4-*Or42b*GAL4); *Or42b* mutant control (*Or42b^−^*; *Or42a*GAL4-*Or42b*GAL4). All GAL4 and UAS lines were described previously (Kreher et al., 2008) or obtained from the Bloomington Stock Center (*Or42b*GAL4: P{Or42b-GAL4.F}64.1). The presence of GAL4 and UAS transgenes in the rescue and control lines were validated using PCR using the following primers: *Or42a*GAL4 (F-GGGCTTTCATTCTTTAGGTC; R-CGATAGAAGACAGTAGCTTC); *Or42b*GAL4 (F-CAAATCGGAAGTCGGGCAACAACA; R-CGATAGAAGACAGTAGCTTC); UAS-*Or42a* (F-GCTTCCAGGACGTTTGCGTTGATT; R-AGTAAGGTTCCTTCACAAAGATC); UAS-*Or42b* (F-TCATCCTTCGTGCTCACTTGGACA; R-AGTAAGGTTCCTTCACAAAGATC).

### Behavioral assays

Odors were obtained from Sigma–Aldrich, at the highest purity possible; odors were dissolved in paraffin oil (Fluka chemical). Each odor was tested in a log_10_ dilution series (volume/volume), ranging from 10^−1^ to 10^−8^. Although odor vapor pressure does not necessarily vary in a linear manner with dilution, testing a range of odor dilutions allows testing of a range of steepness and geometries of gradients. We also note that the same odor concentration experienced in the behavioral arena is not necessarily the same as tested with electrophysiology: for example, 10^−2^ ethyl acetate in the behavioral assay is not completely comparable to ethyl acetate 10^−2^ in the electrophysiological recordings.

Behavioral two-choice assays were conducted to observe chemotaxis of larvae, as described previously (Monte et al., 1989; Kreher et al., 2008). Larvae were placed in the center of a 90 mm plastic dish, coated with 10 ml of 1.1% agarose; 25 µl of dissolved odor were placed on a 1 cm filter paper disk on one end of the plate diameter; 25 µl of paraffin oil alone were placed on a disk at the other end of the diameter. Position of odor versus solvent alone was alternated between behavioral trials (left versus right side of the plate). Approximately 50 early third instar larvae were placed in the center of the plate and were allowed 5 minutes to migrate in the dark. A template was used to define two halves of the plate, and larvae on the odor half versus the control half were then counted, and these counts were used to calculate a response index. The response index can range from 1 to −1, where a value of 1 indicates total attraction and a value of −1 indicates total repulsion. A response index value of 0 represents an equal number of larvae on both sides of the plate, indicating non-chemotaxis.

All behavioral assays were conducted between 10:00 am and 3:00 pm, to reduce variation due to circadian effects. Control and mutant assays were either conducted simultaneously or in series during the same experimental sessions.

### Statistics

Data were analyzed using analysis of variance (ANOVA) using SAS (PROC GLM) (SAS, Cary, NC). The General Linear Model (GLM) procedure was used for ANOVA because it is robust to unbalanced experimental design. The ANOVA model statement included genotype, odor concentration, position of odor on side of plate (left or right), and experimenter. Neither position of odor nor experimenter explained variation by the F statistic. Pairwise means were post-hoc tested by the least squares means test with the Tukey–Kramer test.

## RESULTS

We addressed multiple questions regarding the relationship between odor receptor activity and behavior through examination of chemotaxis behavioral responses of loss-of-function mutants of two odor receptors, *Or42a* and *Or42b*, to three monomolecular ester odors: ethyl acetate, propyl acetate, and ethyl butyrate. The odors used in this study were chosen because both *Or42a* and *Or42b* respond strongly to these odors in the electrophysiological assay, the responses change differentially with change in odor concentration, and additional receptors are recruited to electrophysiologically respond with change in odor concentration (Kreher et al., 2008) (supplementary material Table S1; Fig. S1). Further, because these molecules are found in fruits and plant tissues and are produced in fermentation pathways, they are likely to be ecologically relevant to *Drosophila*. Finally, these three odors are structurally similar and have similar functional groups. We note finally that we are not hypothesizing that *Or42a* and *Or42b* necessarily play an overall important role in chemotaxis behavior, but rather we are using mutants of these odor receptors as tools to test specific hypotheses.

A third odor receptor loss-of-function mutant, *Orco^−^*, was used as a negative control. The *Orco* gene (formerly known as *Or83b*) encodes a broadly expressed odor receptor which is a necessary co-receptor for canonical *Or* receptors and *Orco^−^* larvae are effectively anosmic to almost all odors (Larsson et al., 2004). There are two known additional chemosensory gene families in *Drosophila*, the Gustatory receptors (*Gr*) and Ionotropic receptors (*Ir*), which do not rely on *Orco* (Clyne et al., 2000; Scott et al., 2001; Benton et al., 2009). Thus, using the *Orco^−^* mutation as a negative control allows the identification of odors that only rely on *Or* signaling.

### Non-redundancy among receptors

The first question examined was the individual role of odor receptors that appear redundant in terms of their odor responsiveness. Both *Or42a* and *Or42b* have approximately similar electrophysiological responses (measured in action potentials/s) to the odors propyl acetate and ethyl butyrate at two different tested odor concentrations, 10^−4^ and 10^−2^ (supplementary material Table S1; Fig. S1). Although there are minor differences in electrophysiological responses between the receptors, it is completely unknown how the magnitude of these differences may affect behavioral responses. We began with the question: does *Or42a* or *Or42b* non-redundantly contribute to behavioral responses to propyl acetate or ethyl butyrate? This question was addressed through examining behavioral responses of mutants of *Or42a* and *Or42b* across concentrations of propyl acetate and ethyl butyrate. If *Or42a* and *Or42b* are redundant, relative to any odor receptors, then loss of either receptor should not affect behavior to these odors relative to wild type behavior at any concentration.

**Fig. 1.**
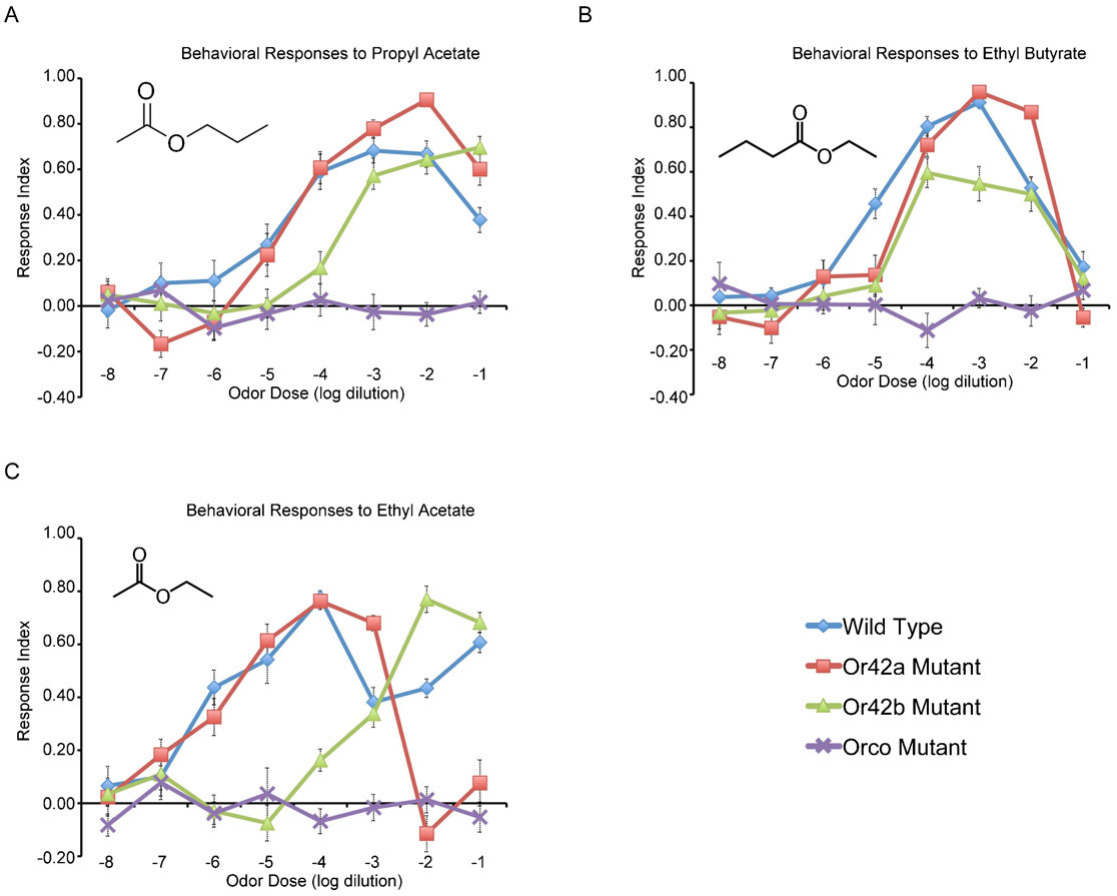
Behavioral responses of odor receptor mutants to odors across concentrations. (A) Behavioral responses of odor receptor mutants to propyl acetate. Inset depicts structural formula of propyl acetate. Error bars represent SEM; n = 9–22 trials for each point. (B) Behavioral responses of odor receptor mutants to ethyl butyrate. Inset depicts structural formula of ethyl butyrate. Error bars represent SEM; n = 10–18 trials for each point. (C) Behavioral responses of odor receptor mutants to ethyl acetate. Inset depicts structural formula of ethyl acetate. Error bars represent SEM; n = 9–26 trials for each point.

Surprisingly, the receptor mutants displayed differences in behavior to these odors at specific concentrations (Fig. 1A,B). While loss of *Or42b* did not affect behavioral responses to propyl acetate at 10^−3^ or 10^−2^ relative to wild type (ANOVA, Tukey–Kramer, p = 1.0000 for each concentration), behavioral responses were reduced at 10^−4^ compared to wild type (ANOVA, Tukey–Kramer, p<0.0001) and elevated at 10^−1^ compared to wild type (ANOVA, Tukey–Kramer, p = 0.0033) (Fig. 1A). Loss of *Or42a* elevated behavioral response to 10^−2^ propyl acetate, although this was not statistically different from wild type (Fig. 1A). Otherwise, the behavioral responses of *Or42a^−^* larvae were approximately similar to wild type. A third receptor, *Or47a*, had a roughly similar electrophysiological response to propyl acetate as *Or42a* and *Or42b* (supplementary material Table S1; Fig. S1), yet *Or42b* is still non-redundant for responses to specific concentrations.

Mutant responses to ethyl butyrate also revealed complex non-redundancy at specific concentrations (Fig. 1B). Whereas loss of *Or42b* reduced behavioral responses to ethyl butyrate at 10^−5^ and 10^−3^ relative to wild type (ANOVA, Tukey–Kramer, p = 0.0017 and p = 0.0065, respectively), responses at other concentrations were not significantly different from wild type. Loss of *Or42a* reduced responses to ethyl butyrate at 10^−5^ relative to wild type (ANOVA, Tukey–Kramer, p = 0.0089), but elevated responses to the odor at 10^−2^ (ANOVA, Tukey–Kramer, p = 0.0007). In conclusion, the differences in electrophysiological responses between *Or42a* and *Or42b* are sufficient to allow non-redundancy in response to specific odor concentrations.

### Behavioral response thresholds

Given that these receptors have non-redundant roles in chemotaxis behavior, it is possible to ask questions at a finer scale. The second question examined was the role of odor receptors in setting behavioral response thresholds. The behavioral response threshold is defined in this study as the lowest concentration of odor where the mean response index of subject larvae is statistically different from the mean response index of negative control larvae, *Orco^−^*; all statistical comparisons reported in this section are relative to *Orco^−^* larvae. The hypothesis for these experiments is that the highest affinity receptors are responsible for determining response thresholds. A high affinity receptor is defined as the receptor of the larval repertoire that gives the highest electrophysiological response at the lowest tested odor concentrations. For the odors ethyl acetate and propyl acetate, *Or42b* is the highest affinity receptor and it should set the behavioral response thresholds; for ethyl butyrate, *Or42a* is the highest affinity receptor, and it should set the behavioral response threshold (supplementary material Table S1; Fig. S1).

Initially, the behavioral response thresholds of wild type larvae were determined (Fig. 1). The response threshold of wild type larvae to propyl acetate was at 10^−4^ (ANOVA, Tukey–Kramer, p<0.0001) (Fig. 1A). The response threshold of wild type larvae to ethyl butyrate was at 10^−5^ (ANOVA, Tukey–Kramer, p = 0.0002) (Fig. 1B). The response threshold of wild type larvae to ethyl acetate was at 10^−6^ (ANOVA, Tukey–Kramer, p<0.0001) (Fig. 1C).

Consistent with the hypothesis, *Or42a* does not play a role in setting the threshold response to propyl acetate or ethyl acetate, revealed through mutation of *Or42a* (Fig. 1A,C). *Or42a^−^* larvae had the same response threshold to propyl acetate at 10^−4^ (ANOVA, Tukey–Kramer, p<0.0001), which is similar to wild type (Fig. 1A). The response threshold of *Or42a^−^* larvae to ethyl acetate was at 10^−6^ (ANOVA, Tukey–Kramer, p = 0.0108), which is similar to wild type (Fig. 1C).

Also consistent with the hypothesis, *Or42b* does play a role in setting the response threshold to both propyl acetate and ethyl acetate (Fig. 1A,C). This was concluded due to the fact that mutation of *Or42b* elevated the response thresholds to these odors. The response threshold of *Or42b^−^* larvae to propyl acetate was increased tenfold to 10^−3^ (ANOVA, Tukey–Kramer, p<0.0001) (Fig. 1A). The response threshold of *Or42b^−^* larvae to ethyl acetate was at 10^−3^ (ANOVA, Tukey–Kramer, p = 0.0003), three orders of magnitude higher than wild type (Fig. 1C).

Reponses to the third tested odor, ethyl butyrate, are partially inconsistent with the hypothesis. While *Or42a* is a higher affinity receptor for ethyl butyrate, both *Or42a* and *Or42b* non-redundantly set the behavioral response threshold to ethyl butyrate (Fig. 1B). The response threshold of *Or42a^−^* larvae to ethyl butyrate was increased tenfold compared to wild type, to 10^−4^ (ANOVA, Tukey–Kramer, p<0.0001). The response threshold of *Or42b^−^* larvae was also at 10^−4^ (ANOVA, Tukey–Kramer, p<0.0001 (Fig. 1B).

In conclusion, while the high affinity receptor typically sets the behavioral response threshold, a complex case was revealed in the analysis of ethyl butyrate, where two receptors can set the threshold in a non-redundant manner.

### Peak responses

The third question examined was how odor receptors set peak responses to odors in the behavioral assay. There are two issues when considering peak response to an odor: the first is the magnitude of the behavioral response; the second is the odor concentration at which the peak occurs. We are defining the peak response magnitude as the highest global response index to any concentration tested within an odor. We are defining the peak odor concentration as the odor concentration at which the peak response occurs.

Our first hypothesis is that the odor receptor with the highest global electrophysiological response determines the magnitude of the peak behavioral response. *Or42a* has the highest overall electrophysiological responses to propyl acetate and ethyl butyrate and should set the peak response magnitude. The case with ethyl acetate is more complex; both *Or42a* and *Or42b* have similar maximal electrophysiological activities but clearly separated dynamic ranges. We have previously partially examined the role of *Or42a* and *Or42b* in setting peak responses to ethyl acetate, but have not comprehensively tested these responses across a broad range of odor concentrations (Kreher et al., 2008).

Consistent with our hypothesis, loss of *O42b* did not decrease the peak response magnitude to propyl acetate; the peak response index of *Or42b^−^* larvae was 0.70, approximately similar to the wild type peak magnitude of 0.68 (Fig. 1A). While loss of *Or42a* did not decrease the peak response index magnitude, loss of *Or42a* did elevate the peak response index to 0.91, compared to the wild type magnitude of 0.68 to propyl acetate (Fig. 1A). Inconsistent with our hypothesis, loss of *Or42b* decreased the peak response magnitude to ethyl butyrate to 0.60, compared to the wild type peak of 0.91 (Fig. 1B). Also inconsistent with our hypothesis, *Or42a^−^* larvae had a peak response index similar to wild type in response to ethyl butyrate, 0.96. In the third case of ethyl acetate, neither loss of *Or42a* nor loss or *Or42b* affected the peak response magnitude (Fig. 1C). Response indices of both mutants to ethyl acetate were approximately similar to wild type.

Does the odor receptor with the highest overall electrophysiological response contribute to the determination of the concentration at which the peak response occurs? The answer appears to be no in each examined case. In each case, *Or42b* contributed to the peak odor concentration, but not *Or42a* (Fig. 1). For propyl acetate, the peak response of *Or42b^−^* larvae was at 10^−1^ instead of the wild type peak between 10^−4^ and 10^−2^ (Fig. 1A). The peak response of *Or42a^−^* larvae to propyl acetate occurred within the same concentration range as wild type (Fig. 1A). For ethyl butyrate, loss of *Or42b* but not *Or42a* affected the odor concentration at which the peak occurred (Fig. 1B). The peak response of *Or42b^−^* larvae to ethyl butyrate occurred at 10^−4^, compared to 10^−3^ of wild type; however, the responses of *Or42b^−^* larvae were similar from 10^−4^ to 10^−2^, such that a single peak did not exist. The peak response of *Or42a^−^* larvae to ethyl butyrate occurred at 10^−3^, similar to wild type (Fig. 1B). Finally for ethyl acetate, loss or *Or42b* shifted the peak response to 10^−2^ compared to 10^−4^ for wild type (Fig. 1C). Loss of *Or42a* did not affect the concentration at which the peak response to ethyl acetate occurred relative to wild type (Fig. 1C).

In summary, the rules for determining peak responses are complex and receptors with highest overall odor responses are not necessarily important for setting peak response magnitude. One receptor, *Or42b*, is necessary for peak response magnitude to ethyl butyrate, whereas loss of *Or42a* increases peak response magnitude to propyl acetate. Receptors with the overall highest electrophysiological responses are also not necessarily important for determining the concentration at which the peak response occurs. In all three cases tested, *Or42b* played a role in determining the peak odor concentration, while *Or42a* was not necessary.

An alternative hypothesis is that the highest affinity receptors are setting peak response magnitudes and concentrations at which they occur. There does not appear to any clear relationship between high affinity receptor and peak response magnitude. The highest affinity receptor may help determine the peak odor concentration of propyl acetate and ethyl acetate, but this hypothesis is not predictive for ethyl butyrate. Responses to ethyl butyrate could represent a special case, or the rules governing peak responses are too complex to make simple conclusions.

### Increased attraction to odors in odor receptor mutants

The fourth question addressed was whether or not odor receptor mutants show increased attraction to odors at any concentrations. In most cases, loss of either *Or42a* or *Or42b* reduced behavioral attraction or had no effect. However, in the previous section, it was noted that loss of *Or42a* and *Or42b* increases attraction to odors at specific concentrations.

There are three cases where loss of *Or42a* increases behavioral attraction to specific odor concentrations. First, as previously stated, loss of *Or42a* increases the response of larvae to 10^−2^ propyl acetate (Fig. 1A). Second, the mean response index of *Or42a^−^* larvae to ethyl butyrate was elevated compared to wild type at 10^−2^ (ANOVA, Tukey–Kramer, p = 0.0007) (Fig. 1B). A third case may be the elevated response or *Or42a^−^* larvae to 10^−3^ ethyl acetate (Fig. 1C), which is elevated above wild type responses (ANOVA, Tukey–Kramer, p = 0.0243). While this difference appears accentuated because of a decreased wild type response to ethyl acetate, the reduced attraction of wild type larvae to ethyl acetate at concentrations above 10^−4^ is consistent with previous findings (Monte el al., 1989; Kreher et al., 2008; Khurana and Siddiqi, 2013).

There are two cases where loss of *Or42b* increases behavioral attraction to specific odor concentrations. First, *Or42b^−^* larvae have elevated responses to propyl acetate at 10^−1^ (ANOVA, Tukey–Kramer, p = 0.0033) (Fig. 1A). Second, responses of *Or42b^−^* larvae to ethyl acetate at 10^−2^ are elevated (ANOVA, Tukey–Kramer, p = 0.0051) (Fig. 1C).

Thus, it appears that some receptors, when mutated, allow an increase in attraction to odors at particular concentrations. One possible explanation for these data is that the nervous system representation via specific receptors at particular odor concentrations causes repulsion and loss of this signal increases attraction. The role of repulsion is a tentative explanation, and our data do not allow us to conclusively state this. Increase of attraction may also be due to gain control in the olfactory circuit, which is discussed later in this report. While we cannot easily determine the coding mechanism underlying these behaviors, these data suggest the mechanisms are very complex.

It is important to note that all of these conclusions can only be made with a comprehensive odor receptor response data set in conjunction with systematic behavior testing. Furthermore, odors must be tested across concentrations, since the roles of odor receptors at each odor concentration are difficult to predict from odor response profiles alone.

### Transgenic rescue of odor receptor mutants

Although the *Or42a* and *Or42b* mutant alleles have been partially previously characterized, the alleles are caused by transposon insertions. To eliminate alternative explanations for the mutant phenotypes, we conducted transgenic rescue experiments, using previously characterized odor receptor GAL4-UAS lines (Kreher et al., 2008). *Or42a* was restored to its native sensory neuron using *Or42a*GAL4-UAS-*Or42a* (*Or42a* rescue) combined with the *Or42a^−^* allele. *Or42b* was restored to its native sensory neuron using *Or42b*GAL4-UAS-*Or42b* (*Or42b* rescue) combined with the *Or42b^−^* allele. Control mutant strains were also constructed by combining either the *Or42a^−^* allele or the *Or42b^−^* allele with *Or42a*GAL4 and *Or42b*GAL4, which should not show rescue and should display the respective mutant phenotypes.

**Fig. 2.**
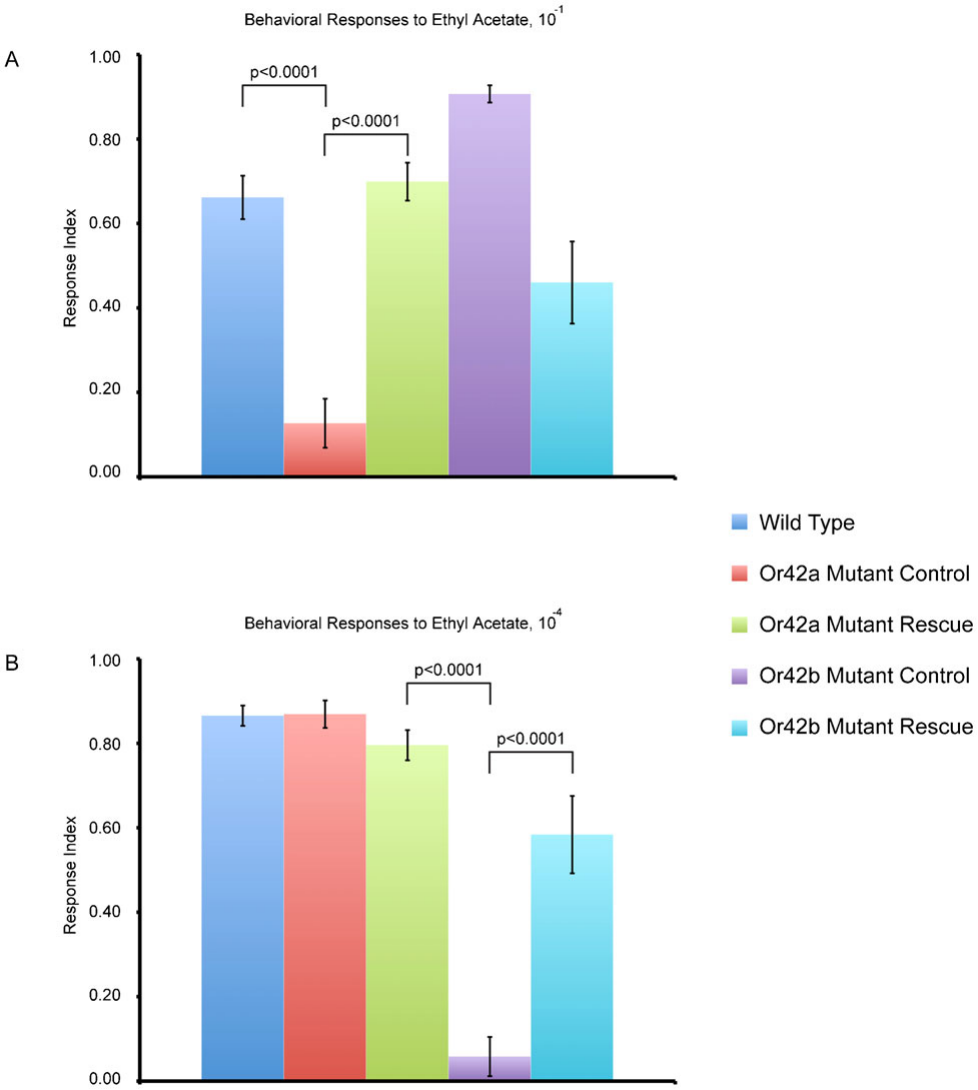
Rescue of odor receptor mutant lines. (A) Behavioral responses of transgenic lines to ethyl acetate, 10^−1^. Mutant controls genotypes are: *Or42a* Mutant Control (*Or42a^−^*; *Or42a*GAL-*Or42b*GAL4) and *Or42b* Mutant Control (*Or42b^−^*; *Or42a*GAL4-*Or42b*GAL4). Rescue genotypes are: *Or42a* Mutant Rescue (*Or42a^−^*; *Or42a*GAL4-UAS-*Or42a*) and *Or42b* Mutant Rescue (*Or42b^−^*; *Or42b*GAL4-UAS*Or42b*). Error bars represent SEM. n = 5–11 trials. (B) Behavioral responses of transgenic lines to ethyl acetate, 10^−4^. Genotypes are same as listed previously. Error bars represent SEM. n = 8–11 trials.

Control mutant *Or42a^−^* larvae had greatly reduced behavioral attraction to 10^−1^ ethyl acetate compared to wild type as expected (ANOVA, Tukey–Kramer, p<0.0001) (Fig. 2A). Rescue of *Or42a* using GAL4-UAS restored behavioral attraction to 10^−1^ ethyl acetate to approximately wild type levels, and responses were significantly different from mutant responses (ANOVA, Tukey–Kramer, p<0.0001) (Fig. 2A). Control mutant *Or42b^−^* larvae also showed slightly elevated attraction to 10^−1^ ethyl acetate, consistent with previous trends. Rescue of *Or42b* also showed approximately wild type behavioral responses to 10^−1^ ethyl acetate, as expected (Fig. 2A).

As expected, control *Or42a^−^* larvae showed wild type levels of attraction to 10^−4^ ethyl acetate (Fig. 2B). Rescue of *Or42a* in response to 10^−4^ ethyl acetate was also equivalent to wild type levels as expected. Control mutant *Or42b^−^* larvae matched prediction by showing strongly reduced attraction to 10^−4^ ethyl acetate compared to wild type (ANOVA, Tukey–Kramer, p<0.0001) (Fig. 2B). Finally, rescue of *Or42b* elevated attraction to 10^−4^ ethyl acetate. The behavioral response of *Or42b^−^* larvae was not completely at wild type levels, but was significantly elevated compared to the control mutant (ANOVA, Tukey–Kramer, p<0.0001) (Fig. 2B).

### Vapor pressures of odor molecules do not fully explain response thresholds and peak responses

In the two-choice behavioral assay, the different tested odors elicited threshold responses and peak responses that occurred at concentrations that varied by orders of magnitude. One question with this assay however, is the extent to which vapor pressures of odor molecules determine this variation in responses. For example, do molecules with higher vapor pressures elicit lower behavioral response thresholds and peak responses at lower odor concentrations? In the two-choice assay, the odor concentration gradient occurs through diffusion and the geometry and steepness of the gradient is unknown, compared to other assays with defined gradients (Gomez-Marin et al., 2011; Gershow et al., 2012; Gomez-Marin and Louis, 2014). Alternatively, how much variation in behavioral responses is determined by odor receptor activity in addition to vapor pressure? For example, ethyl acetate elicits the lowest behavioral threshold from wild type larvae at 10^−6^, which is at least an order of magnitude lower than the threshold responses to propyl acetate and ethyl butyrate. One explanation for this is a single odor receptor (*Or42b*) has a higher global electrophysiological response to ethyl acetate at the lowest tested odor concentration than any receptor's response to propyl acetate or ethyl butyrate. However, the vapor pressure of ethyl acetate is 4–6 fold higher than either propyl acetate or ethyl butyrate. Does vapor pressure explain variation in measured behavioral values such as response threshold or peak response concentration?

**Fig. 3.**
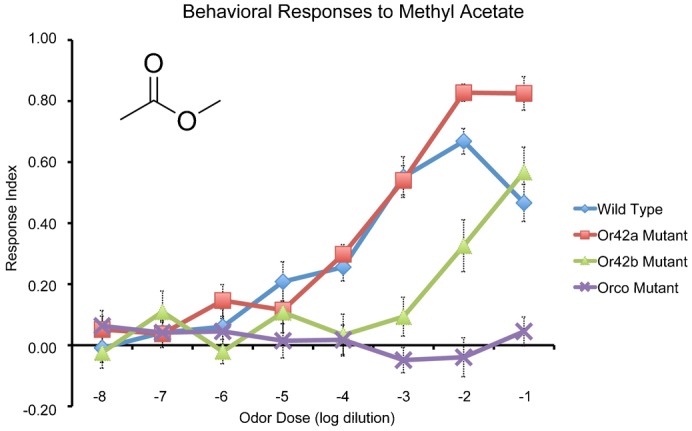
Behavioral responses of odor receptor mutants to methyl acetate. Inset depicts structural formula of methyl acetate. Error bars represent SEM; n = 10–16 trials for each point.

To address this question, we chose to study behavioral responses of the mutant panel to a fourth odor, methyl acetate (Fig. 3). Methyl acetate was chosen because of its similar structure and functional groups compared to the other three esters. However, the vapor pressure of methyl acetate is 169 mmHg, versus 95 mmHg for ethyl acetate, 25 mmHg for propyl acetate, and 15 mmHg for ethyl butyrate, all at 25°C (Haynes et al., 2013). Thus, if high vapor pressure predominately determines behavioral response variation in accordance with the trends observed, methyl acetate should have the lowest response threshold and the peak responses should occur at a relatively lower odor concentration.

The response threshold of wild type larvae to methyl acetate was at 10^−3^ (ANOVA, Tukey–Kramer, p<0.0001) (Fig. 3). Interestingly, although methyl acetate has the highest vapor pressure of the four tested odors, it does not have the lowest response threshold.

Did methyl acetate have a higher peak response magnitude compared to the other esters? The peak response magnitude to methyl acetate was 0.67 (Fig. 3), which is approximately similar to the peak response index to propyl acetate of 0.68 (Fig. 1A), but lower than the peak response index to ethyl butyrate of 0.91 (Fig. 1B).

Did the peak response index to methyl acetate occur at lower odor concentrations than the other esters? The peak response index to methyl acetate occurred at 10^−2^ (Fig. 3), which is tenfold higher than the peak concentration of ethyl butyrate, and two orders of magnitude higher than the peak concentration of ethyl acetate (Fig. 1C).

Thus, there is no clear pattern between vapor pressure and behavioral response threshold, peak response magnitude, or the peak response concentration. Odors with high vapor pressures do not necessarily have low behavioral response thresholds, nor do they have larger peak response magnitudes, nor do peak responses occur at lower odor concentrations.

The next question addressed was the role of *Or42a* and *Or42b* in behavioral responses to methyl acetate. The response threshold of *Or42a^−^* larvae was similar to wild type larvae, also at 10^−3^ (ANOVA, Tukey–Kramer, p<0.0100) (Fig. 3). The peak response magnitude of *Or42a^−^* larvae was 0.83, which is elevated above wild type larvae. The peak response concentration was between 10^−2^ and 10^−1^ (Fig. 3).

By contrast, the response threshold of *Or42b^−^* larvae was increased compared to wild type by tenfold to 10^−2^ (ANOVA, Tukey–Kramer, p = 0.0008) (Fig. 3). The peak response magnitude of *Or42b^−^* larvae was approximately similar to wild type and the peak response concentration was shifted tenfold higher relative wild type, to 10^−1^ (Fig. 3).

### Loss of *Or42a* affects behavioral responses to odors that are not strong electrophysiological ligands

While we behaviorally tested three esters (propyl acetate, ethyl butyrate, and ethyl acetate), which strongly activate *Or42a* and *Or42b* in the electrophysiological assay, we also tested three odors, which are not strong activators of either receptor. Neither acetophenone, anisole, nor 3-octanol activates *Or42a* or *Or42b* above the 50 action potentials/s threshold (supplementary material Table S1; Fig. S1). We note that although *Or42a* and *Or42b* are not strongly activated by these three odors, other odor receptors are strongly activated at these concentrations. To examine whether *Or42a* or *Or42b* play a role in behavioral responses to non-activating odors, we tested the odor receptor mutants in the two-choice chemotaxis assay.

**Fig. 4.**
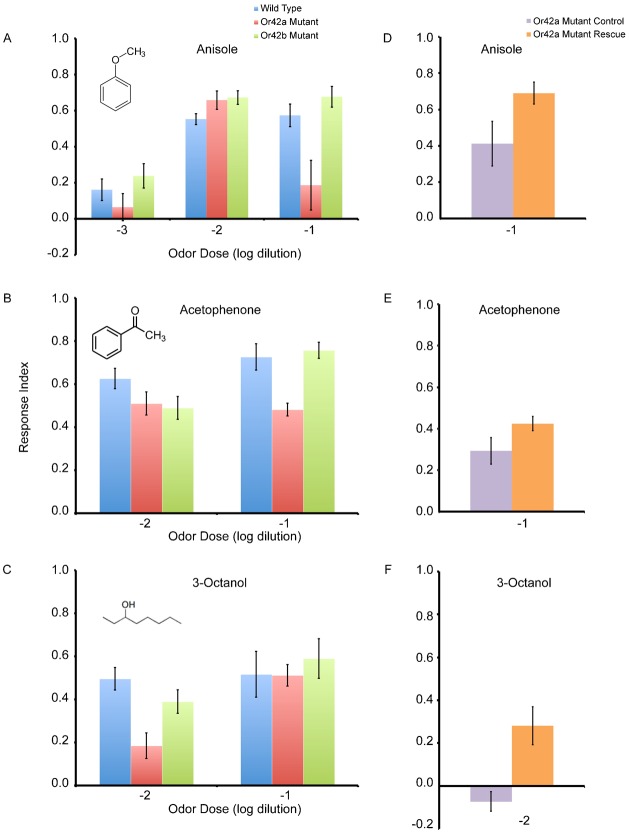
Behavioral responses to modest activators of *Or42a* and *Or42b*. (A) Behavioral responses to anisole, 10^−1^ to 10^−3^. Inset shows structural formula of anisole. (B) Behavioral responses to acetophenone, 10^−1^ and 10^−2^. Inset shows structural formula of acetophenone. (C) Behavioral responses to 3-octanol, 10^−1^ and 10^−2^. Inset shows structural formula of 3-octanol. (D) Rescue or *Or42a* in response to anisole, 10^−1^. *Or42a* Mutant Control genotype: (*Or42a^−^*; *Or42a*GAL-*Or42b*GAL4). *Or42a* Mutant Rescue genotype: (*Or42a^−^*; *Or42a*GAL4-UAS-*Or42a*). (E) Rescue or *Or42a* in response to acetophenone, 10^−1^. Genotypes same as listed previously. (F) Rescue or *Or42a* in response to 3-octanol, 10^−2^. Genotypes same as listed previously. For all experiments, error bars represent SEM; n = 6–14 trials.

Surprisingly, the *Or42a^−^* mutant has decreased behavioral attraction to the 10^−1^ concentration of anisole compared to wild type (ANOVA, Tukey–Kramer, p = 0.0103) (Fig. 4A). We also note that the behavioral responses of *Or42a^−^* mutant larvae are much more variable compared to wild type, seen in the increase of SEM value relative to the other genotypes. The effect of the *Or42a^−^* mutant is specific to 10^−1^ anisole, because responses of the mutant are similar to wild type at 10^−2^ and 10^−3^. The response of the *Or42b^−^* mutant is not affected and is similar to wild type to anisole at 10^−1^ to 10^−3^ (Fig. 4A).

The *Or42a^−^* mutant also has decreased response to the 10^−1^ concentration of acetophenone (Fig. 4B). The effect is specific, because the mutant does not affect behavioral response to acetophenone 10^−2^. The response of the *Or42b^−^* mutant is similar to wild type at both 10^−1^ and 10^−2^.

Behavioral responses to a third odor, 3-octanol 10^−1^ are similar between wild type and both *Or42a^−^* and *Or42b^−^* mutants (Fig. 4C). However, responses of *Or42a^−^* larvae to 3-octanol at 10^−2^ are reduced compared to wild type controls. Responses of *Or42b^−^* larvae are similar to wild type at both concentrations of 3-octanol.

Are these subtle phenotypes truly due to odor receptor mutants or other causes? We tested the *Or42a* rescue line and mutant control line at specific odor concentrations (Fig. 4D–F). While there was some variation in the control mutant line in response to these odors, the *Or42a* rescue elevated behavioral attraction to these odors in each case. Rescue did not restore behavioral response to wild type levels in each case, indicating possible presence of interacting alleles. Additionally, the *Or42a* rescue responses did not reach statistical difference compared to control lines. However, the trend clearly indicates elevation of attraction to each odor in the rescue lines.

Why is *Or42a* playing a role in behavioral responses to high concentrations of anisole, acetophenone, and 3-octanol? Revisiting the electrophysiological data, 10^−2^ acetophenone elicits a mean response of 36 action potentials/s, 10^−2^ anisole elicits a mean response of 25 action potentials/s, and 10^−2^ 3-octanol elicits a mean response of 27 action potentials/s from *Or42a* (supplementary material Table S1; Fig. S1). However, responses to paraffin oil alone and spontaneous activity have been subtracted to yield those means. The raw mean response of *Or42a* to 10^−2^ acetophenone is 75 action potentials/s, to 10^−2^ anisole is 63 action potentials/s, and to 10^−2^ 3-octanol is 66 action potentials/s, relative to a response of *Or42a* to paraffin oil of 39 action potentials/s and a spontaneous rate of action potentials of 13 action potentials/s. Although the responses of *Or42a* to anisole, acetophenone, and 3-octanol are only modestly elevated compared to controls, these differences may be enough to contribute meaningfully to allow odor coding. Additionally, responses to these odors may increase differentially as odor concentration is increased.

## DISCUSSION

### The rules by which odor receptors allow odor coding

Deducing the rules by which odor receptors allow coding has been extremely difficult without comprehensive descriptions of how a full repertoire of receptors respond to odors coupled with systematic behavioral testing. While it was fully reasonable to predict that the rules of odor coding are complex prior to any empirical analysis, results from systematic behavioral testing have revealed that we can reasonably predict some aspects of behavior while we can only eliminate some other factors as being explanatory.

Behavioral response thresholds appear to be determined by the highest affinity receptors of the repertoire, while the rules that govern peak responses in the two-choice assay are less clear. Peak responses to do not necessarily rely on receptors with the highest global electrophysiological responses; high affinity receptors may determine peak responses, but for one comprehensively tested odor this is not the case.

One problem with prediction of behavior from electrophysiological receptor activity is that many receptors appear redundant with respect to odor response. This is particularly true of animals with hundreds of receptors. Responses of the larval receptor repertoire to propyl acetate and ethyl butyrate are respectively very similar, yet by mutation analysis the individual receptors do play unique roles at specific concentrations in mediating behavior (Fig. 1). A similar observation has been made of mouse odor receptors, where some Trace-amine associated receptors (TAARs) play non-redundant roles in behavioral responses to amines (Dewan et al., 2013). By Mill's method of residues, we can deduce that residual behavioral attraction to odors, upon mutation of an individual receptor, is most likely due to activity of remaining odor receptors. For example, upon loss of *Or42b*, behavioral attraction to propyl acetate appears to shift tenfold at every odor concentration. Other responsive receptors, such as *Or35a*, *Or42a*, and *Or47a*, may explain the remaining attraction. One method of exploring this hypothesis further is to examine double-mutants of these odor receptors.

It may be tempting to speculate that loss of odor receptors shifts perception of odors. For example, the behavioral responses of *Or42b^−^* larvae appears shifted tenfold in response to propyl acetate at every concentration (Fig. 1A) and shifted two orders of magnitude in response to ethyl acetate at every concentration (Fig. 1C). However, the meaning of “shift of perception” is difficult to define, and much more analysis is required to understand the meaning and implications of this.

How can receptors with similar electrophysiological responses across concentrations of an odor have non-redundant roles in odor coding? Although we have only considered a simple action potential rate code, odor receptors display a large diversity of response kinetics and adaptation (de Bruyne et al., 2001; Hallem and Carlson, 2006). Ultra-sustained responses of odor receptors can be elicited by specific odors and these responses can play a role in odor coding in both *Drosophila* and *Anopheles* (Montague et al., 2011; Turner et al., 2011). The electrophysiological responses of *Or42a* and *Or42b* to the tested panel of odors do show diverse kinetics, which vary with odor concentration (data not shown). Consideration of these properties may allow a better explanation of how a receptor can generate non-redundant signals.

A general emerging theme is that odors are coded in a weak combinatorial manner, where some receptors and sensory neurons may be more important than others. In this study, *Or42b* was necessary for setting all peak response concentrations and was non-redundantly necessary for the threshold response to ethyl butyrate, despite not being the highest affinity receptor. Consistent with this conclusion, a recent study of larval odor receptors found that the odor acetal, which elicits electrophysiological activity only in *Or42b*, elicits the strongest behavioral response of a tested panel of odors, whereas odors that elicit strong activity from other receptors do not elicit large chemotaxis behavioral responses (Mathew et al., 2013). Furthermore, mutations in odor receptors do not necessarily cause alterations in behavior in response to odors which are electrophysiological ligands (Elmore et al., 2003; Keller and Vosshall, 2007). In a recent study of adult *Drosophila* glomerular responses, although multiple glomeruli responded to the odor mixture, apple cider vinegar, only a subset of the respective olfactory sensory neurons classes were necessary for attraction to the odor mixture, whereas others were redundant or not necessary for behavior (Semmelhack and Wang, 2009).

Another important observation in these data is that some odor receptor mutants displayed increased attraction at particular odor concentrations. One explanation is that the sensory representation of particular odor concentrations via specific receptors causes a repulsive effect, overlaid on attractive signals generated by other receptors. If the repulsive signals are lost, the remaining attractive signals increase behavioral response. This is a speculative explanation and much further work is required to make a stronger conclusion. A second explanation for these data may be that second order local interneurons in the antennal lobe may modulate output of incoming sensory information to allow gain control of the circuit. Asahina et al. found that engineering larvae with single functional olfactory receptor neuron classes had altered responses to odors compared with wild type larvae, and that local interneurons in such animals also needed multiple functional olfactory receptor neurons to show odor-evoked activity (Asahina et al., 2009). Our results are partially compatible with this model: while loss of *Or42a* reduces responses to high concentrations of ethyl acetate, in other cases loss of *Or42a* increases attraction to specific odor concentrations. This may imply that local interneurons have functional diversity, where some serve to dampen responses to high odors and others allow differential transmission of sensory information. A second possibility is that local interneurons are not activated by all odors, so that their filtering function could be odor and concentration specific. Gain control of projection neurons is also an explanation consistent with behavioral alterations in response to weak activators of *Or42a*, and is considered in more detail later.

One limitation of the electrophysiological data set is that it was derived from odor receptors analyzed in the “empty neuron” system. In many cases activities in the empty neuron match responses from native olfactory sensory neurons (Hallem et al., 2004; Jones et al., 2007). Odor-binding proteins and other signaling molecules also contribute to shaping sensory neuron responses (Benton et al., 2007; Laughlin et al., 2008), and taking these factors into account will lead to more confident conclusions about the sensory representations of odors.

While our results apply to probability of chemotaxis behavior, analysis of other behaviors or quantitative analysis of subsets of behavior, such as velocity or turning probability, may reveal expanded roles for *Or42a* in odor-mediated behavior. While the responses of odor receptor repertoires have overall explanatory power for odor segmentation behavior and odor discrimination behavior (Kreher et al., 2008; Parnas et al., 2013), the role of individual receptors in these behaviors is unknown. One interesting direction would be to test odor-discrimination behavior of *Or42a^−^* larvae.

### Vapor pressures and behavioral response threshold and peak response

In the two-choice assay, the odor gradient is established by passive diffusion of odor molecules, whereas other assays allow active establishment of gradients of known geometries and steepness (Gomez-Marin et al., 2011; Gershow et al., 2012; Gomez-Marin and Louis, 2014). In the two-choice assay, does vapor pressure of the molecule explain behavioral response thresholds and peak responses? For example, in the two-choice assay, ethyl acetate has a behavioral response threshold at 10^−6^ versus a response threshold of propyl acetate at 10^−4^. Can this difference be explained by the vapor pressure of ethyl acetate, which is fourfold higher than the vapor pressure of propyl acetate? Or, is this observation dependent on the observation that an odor receptor has an elicited electrophysiological response to ethyl acetate that is twofold higher than propyl acetate at the lowest tested odor concentration? By testing a molecule, methyl acetate, with an even higher vapor pressure than ethyl acetate, it appears that vapor pressure cannot fully explain threshold response or peak response concentration in the two choice assay.

What roles do the odor receptors *Or42a* and *Or42b* play in behavioral responses to methyl acetate? *Or42b* appears to be necessary for determining both the behavioral response threshold and the peak response concentration, whereas loss of *Or42a* does not affect behavioral response threshold but does elevate peak response magnitude (Fig. 3). While methyl acetate was not included in the electrophysiological panel, it will be useful to systematically test responses to this odor. However, the mutant phenotypes are tentative evidence underlying the differential weighting of *Or42b* in behavioral responses relative to *Or42a*.

### Modest odor receptor responses are important for behavior

A puzzling observation presented here is that loss of *Or42a* affects behavioral responses to odors that elicit small/modest electrophysiological responses. How may small/modest elicited electrophysiological responses be relevant for behavior? One explanation is that these small responses are sufficient to elicit activity in the projection neuron post-synaptic to the *Or42a* sensory neuron.

A second, non-mutually exclusive, explanation is that the modest responses to acetophenone, anisole, and 3-octanol cause pre-synaptic lateral inhibition of other projection neurons through local interneurons, allowing gain control in projection neurons. As explained in the previous section, at least two types of local interneurons exist in the antennal lobe: excitatory cholinergic and inhibitory GABAergic (Wilson et al., 2004; Shang et al., 2007; Olsen and Wilson, 2008; Olsen et al., 2010). While both excitatory and inhibitory responses play a role in shaping projection neuron response, inhibitory responses may predominate (Olsen et al., 2010). Projection neurons tend to saturate more quickly and at relatively modest sensory neuron responses; inhibition of GABA receptors accentuates this saturation, indicating a role of GABAergic neurons in allowing projection neurons to remain responsive across odor concentrations (Wilson et al., 2004; Olsen et al., 2010). The loss of *Or42a* may interfere with gain control by preventing lateral inhibition of projection neurons, causing projection neurons to be saturated at higher odor concentrations. A prediction of this explanation is that behavior, due to altered projection neuron responses, should only be affected at high, but not low, odor concentrations, which has been observed in our study (Fig. 4). Although most studies have focused on the adult antennal lobe, local interneurons are present in the larval antennal lobe, and the same principles may apply (Ramaekers et al., 2005; Asahina et al., 2009).

In a recent analysis of larval behavior, ectopic expression of *Or42a* in multiple sensory neurons affected behavioral responses to both 3-octanol and anisole. The proposed explanation for these results was that ectopic *Or42a* expression was affecting receptor function in non-native sensory neurons (Tharadra et al., 2013). A second explanation, consistent with the data presented here, is that modest electrophysiological responses of *Or42a* to these odors is altering gain control, thereby altering behavioral responses.

The anatomical and numerical simplicity of the *Drosophila* larval olfactory system belies a complexity of odor coding. A first major conclusion is that we have elucidated some rules by which odor receptors allow behavioral responses to odors. In other more complex cases, we have eliminated some possible explanations for some behaviors. A second major conclusion is that modest electrophysiological odor receptor responses may be important as well, and may influence behavior via lateral inhibition. An important future direction will be to test the odor receptor mutants in additional behavioral assays, such as tests of odor segmentation and odor learning. It may be that different odor-mediated behaviors are coded by different olfactory sensory channels: for example, while *Or42b* appears to be crucial for chemotaxis behavior, *Or42a* may play a role in determination of odor identity. The hypotheses and evidence generated by studying the *Drosophila* larva can be used to address questions in animals with more complex olfactory systems.

## Supplementary Material

Supplementary Material
