# Supplementary material for: Complex and non-redundant signals from individual odor receptors that underlie chemotaxis behavior in Drosophila melanogaster larvae
Source: Biol Open. 2014 Sep 19;3(10):947–57. doi: 10.1242/bio.20148573 (PMC4197443; doi:10.1242/bio.20148573)
Supplement: Supplementary Material [file supp_3_10_947__index.html]

Complex and non-redundant signals from individual odor receptors that underlie chemotaxis behavior in Drosophila melanogaster larvae — Complex and non-redundant signals from individual odor receptors that underlie chemotaxis behavior in Drosophila melanogaster larvae — Supplementary Material 

# Complex and non-redundant signals from individual odor receptors that underlie chemotaxis behavior in *Drosophila melanogaster* larvae

## bio.20148573 Supplementary Material

**Files in this Data Supplement:**

- Supplementary Material - Jeewanjot S. Grewal et al. doi: 10.1242/bio.20148573
