# Supplementary material for: Complex and non-redundant signals from individual odor receptors that underlie chemotaxis behavior in Drosophila melanogaster larvae
Source: Biol Open. 2014 Sep 19;3(10):947–57. doi: 10.1242/bio.20148573 (PMC4197443; doi:10.1242/bio.20148573)
Supplement: Supplementary Material [file supp_bio.20148573_bio.20148573-s1.pdf]

Supplementary Material
Jeewanjot S. Grewal et al. doi: 10.1242/bio.20148573

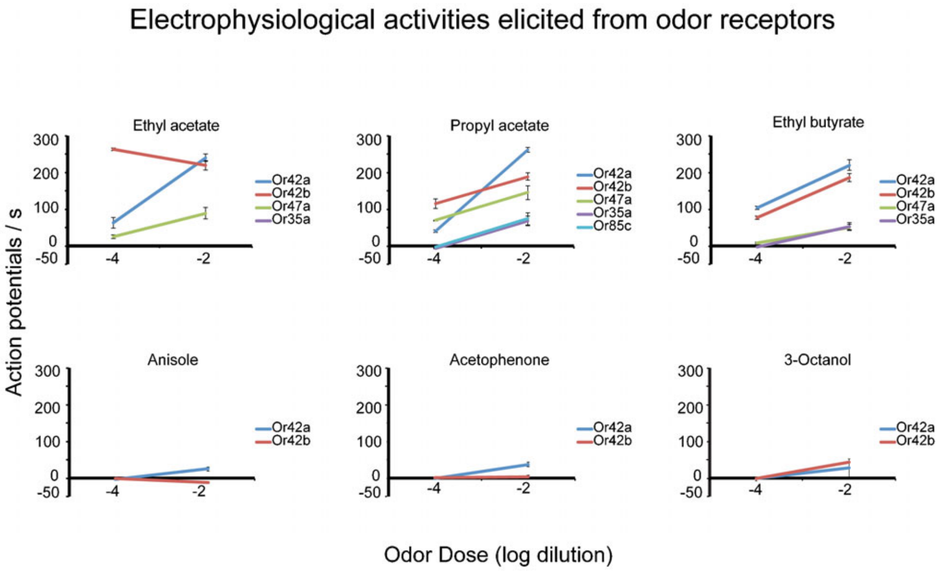

Fig. S1. Re-represented data from supplementary material Table S1. Electrophysiological responses of selected larval odor receptors to selected odors are plotted in action potentials/s, by odor dose (10^{-2} and 10^{-4}). Action potential rates were only quantified during the 500 ms odor stimulation period. Error bars represent SEM. Adapted from Kreher et al., 2008 (Kreher et al., 2008).

Biology Open (2014) 0, 1–11 doi:10.1242/bio.20148573

Table S1. Electrophysiological responses of larval odor receptors to selected odors

	Or2a	Or7a	Or13a	Or22c	Or24a	Or30a	Or33b	Or35a	Or42a	Or42b	Or45a	Or45b	Or47a	Or49a	Or59a	Or67b	Or74a	Or82a	Or85c	Or94a	Or94b
**10^{-2} dilution**																					
ethyl acetate	.	.	–	–	.	.	.	.	++++	++++	.	.	+	.	.	.	.	.	.	.	.
ethyl butyrate	.	.	.	–	–	.	.	+	++++	+++	.	.	+	.	.	.	.	.	.	.	.
propyl acetate	.	.	.	.	.	.	–	+	++++	+++	+	.	++	.	.	.	.	.	+	.	.
anisole	.	–	–	+	++++	++++	–	–	.	–	.	++	.	.	++	+	.	.	.	++++	.
acetophenone	.	.	–	++	++++	++	.	–	.	.	.	++++	.	.	+	++++	.	.	–	.	.
3-octanol	.	.	++	–	–	.	–	+	.	.	+	.	++	.	.	.	.	.	++++	.	.
**10^{-4} dilution**																					
ethyl acetate	.	.	.	.	.	.	–	–	+	++++	.	.	.	.	.	.	–	–	–	.	.
ethyl butyrate	.	.	.	.	.	.	–	.	++	+	.	.	.	.	.	.	.	.	–	.	.
propyl acetate	.	.	.	.	.	.	–	–	.	++	.	.	+	.	.	.	.	.	–	.	.
anisole	.	.	.	–	.	.	–	.	.	.	.	.	.	.	.	.	.	.	.	.	.
acetophenone	.	–	.	.	++++	.	–	.	.	.	.	+	.	.	.	+	.	.	.	.	.
3-octanol	.	.	.	.	.	.	–	.	.	.	.	.	.	.	.	.	.	.	++	.	.

Summary of action potential rates elicited from odor receptors tested in the empty neuron system. "." <50 action potentials/s, "+" = between 50 and 100 action potentials/s, "++" = between 100 and 150 action potentials/s, "+++" = between 150 and 200 action potentials/s, "++++" >200 action potentials/s. Action potential rates were only quantified during the 500 ms odor stimulation period. Adapted from Kreher et al., 2008 (Kreher et al., 2008).
